# Psychography

**Published:** 1888-02

**Authors:** 


### PSYCHOGRAPHY.

### A PROVEN FACT.

Hearing from a friend that a spirit medium had recently come to the Hub from the roaring wilds of Michigan, who was by spirit power or by sleight-of-hand outdoing the famed Kellar or Hermann, I asked: "Can he equal Slade, Bishop, Montague, or the once famous Foster?" "Oh!" he replied, "I tell you he can knock Slade and Foster out the first round, and as for Bishop and Montague, he can do them up before he starts. On my soul," exclaimed my excited friend, "he summons the spirits from the vasty deep by dozens. I have just had a sitting with him. Why, sir, I got a long communication from my brother, in his own handwriting; also, one from my mother and our old preacher. These communications were written between two slates, which I held while the writing was being done."

Satisfied that my friend was off his base, or that he was a victim of a trick, I noted down carefully the name and address, and proceeded directly to the place for the purpose of investigating the spirit claim, or rather exposing, as I have frequently done, a trickster.

In twenty minutes I was at 109 Falmouth street, an apartment house just off Chester Park, near Huntington Avenue. I touched the electric button. The door was opened. I ascended one flight; was met at the door by a little boy, who, having but one eye, looked as though he might see like a spirit out of the other.

"Is this where Mr. Watkins lives?" I asked. "Yes," was the reply. "I mean C. E. Watkins." "Yes." "Is he in?" "Yes." "Can I see him?" "Yes."

I was ushered into the reception-room. In a few minutes Mr. Watkins entered. I was surprised at seeing such a fine-looking man, a man of fine brain, pleasing manners, an honest face, and seemingly well educated, and, as I have since learned a cousin of the novelist Howells.

"Are you the medium who gives sittings for independent slate writings?" "I am," was the prompt reply. "Can I have one?" "You can."

At this I produced four slates.

"You can write the names of several of your departed friends on a slip of paper, and ask each one a question; then fold the slips into little balls. I will return in a few minutes."

I wrote the names of nine different persons who were dead, asking each one a question, rolled each slip, which contained a name and question, into a fine ball, and when done I could not tell which was which. I had read the report of the Seybert commission, how they claimed that the slate-writing was done by the medium with his feet, and I prepared myself for the trick. In a few moments Mr. Watkins entered.

"Point your pencil toward the little paper balls," he said.

I did so, he standing off three or four feet from me.

"Pick up that one," he said; "it contains the name of a lady; her name is (I will give the initials only) E. G.; she says her middle name is C., which you have forgotten; but, as you were an old lover, you can look at some of her letters, which are in your vault of the Safety Deposit Vaults of this city, if you do not already remember. She also says the last time she saw you was at Trenton, N. J., and you promised——"

"Hold on," I said, "don't you give secrets out of school."

"This spirit says," continued Mr. Watkins, "if you will take up two of the slates she will write you a communication between them, with nobody touching them but yourself ; that your father, who died in Chester, Penn., four years ago, will also write a communication."

I picked up the slates and instantly heard something writing between them. In less than half a minute the writing ceased, and there were two communications filling both sides of the slate, one in the handwriting of the young lady and the other in the exact handwriting of my father.

"Take up the other slates," he said, and in less than a minute, in the same way, I got three different communications, and one from my little girl who had been dead nearly a year, written in her broken writing, and talking just as she did.

"Look on the other side of the slate," said the medium.

I did, and there was a perfect picture of my little girl, wearing the winter hood she wore the last time she was upon the street.

"This picture work of the spirits," Mr. Watkins said "seldom occurs."

All the rest of my questions were then answered. So thoroughly astounded and almost paralyzed was I, that I left without expressing myself to the medium. In just one hour I was back with our old Judge and a brother lawyer. The same performance of writing names being over, the Judge said :

"Now, Mr. Medium, trot out your spirits, if you have got any. I bet you $100 you can't get any writing between these slates, if you keep your own fingers off of them."

"Take your slates," said the medium, "into the other room, and sit down on them."

This the Judge, though inclined to be fat, did in a most simple manner.

"Now get up," said the medium, "and open your slates."

He did so, and there were two full communications filling both sides of the slates, and signed, the one from a prominent lawyer, the other from a book publisher, both well known in Boston, and only dead about two and five years ago. The handwriting was exactly their handwriting.

Over forty persons have examined these communications, and pronounced the writing and signatures genuine. Mr. Watkins is busy all the time with those seeking an interview with their friends from the vast unknown. His charges are $3, $5 and $10, according to the length and character of the sitting. I have had seven sittings, each one being more wonderful than the preceding one. I have only given an outline of a few things which occurred. If this is not done by spirit power, will some of the wise men and scientists of Boston explain how it is done ?—*Boston Daily Globe.*

---

Mice in Orchards.—I will give you an infallible preventive which will cost nothing but a little work. Late in the season, before the ground is frozen, cut out all grass near the trunks of your trees, with a sharp hoe, then shovel up to them clean soil, hilling up somewhat, and to extend a foot or more around the trees, and pack with shovel or trample with feet, solid. Mice will then find no harbor next the trees, nor will they injure them in any way. Try this sure remedy, and feed your oats to your horses.—*Vick's Magazine* for December.

